# Investigation of Cross-Contamination and Misidentification of 278 Widely Used Tumor Cell Lines

**DOI:** 10.1371/journal.pone.0170384

**Published:** 2017-01-20

**Authors:** Yaqing Huang, Yuehong Liu, Congyi Zheng, Chao Shen

**Affiliations:** 1 State Key Laboratory of Virology, College of Life Science, Wuhan University, Wuhan, Hubei, China; 2 China Center for Type Culture Collection, Wuhan, Hubei, China; Centro de Investigacion y de Estudios Avanzados del Instituto Politecnico Nacional, MEXICO

## Abstract

In recent years, biological research involving human cell lines has been rapidly developing in China. However, some of the cell lines are not authenticated before use. Therefore, misidentified and/or cross-contaminated cell lines are unfortunately commonplace. In this study, we present a comprehensive investigation of cross-contamination and misidentification for a panel of 278 cell lines from 28 institutes in China by using short tandem repeat profiling method. By comparing the DNA profiles with the cell bank databases of ATCC and DSMZ, a total of 46.0% (128/278) cases with cross-contamination/misidentification were uncovered coming from 22 institutes. Notably, 73.2% (52 out of 71) of the cell lines established by the Chinese researchers were misidentified and accounted for 40.6% of total misidentification (52/128). Further, 67.3% (35/52) of the misidentified cell lines established in laboratories of China were HeLa cells or a possible hybrid of HeLa with another kind of cell line. Furthermore, the bile duct cancer cell line HCCC-9810 and degenerative lung cancer Calu-6 exhibited 88.9% match in the ATCC database (9-loci), indicating that they were from the same origin. However, when we used 21-loci to compare these two cell lines with the same algorithm, the percent match was only 48.2%, indicating that these two cell lines were different. The SNP profiles of HCCC-9810 and Calu-6 also revealed that they were different cell lines. 150 cell lines with unique profiles demonstrated a wide range of *in vitro* phenotypes. This panel of 150 genomically validated cancer cell lines represents a valuable resource for the cancer research community and will advance our understanding of the disease by providing a standard reference for cell lines that can be used for biological as well as preclinical studies.

## Introduction

Although cell line authentication has been widely recommended for many years, misidentification, including cross-contamination, remains an unresolved issue [1, 2]. With the development of biomedical research, more and more scientific reports involving human cell lines have been published by researchers in China. However, authentication has been neglected by many researchers [3]. Until recently, many major journals and research agencies recommended that cell lines should be verified for authenticity before publication or inclusion in grant applications. Although there are many new methods for cell line authentication [4, 5], DNA profiling based on short tandem repeat (STR) is still proposed as a gold standard method. Our lab started using STR for cell line authentication from 2009 with 16/20-loci STR and found about 25% cross-contamination or misidentification among 380 cell lines from 2009 to 2013 [6]. In this study, we provided 21-loci STR for 278 cancer cell lines (data collected from 2014 to 2016), in accordance with the major cell repository’ recommendation for the testing of cell line identity. We observed examples of cross-contamination and misidentification of cell lines, and provided reference STR profiles for cancer cell lines that are currently unavailable in the STR database of major cell repositories. Additionally, we have extended the number of genetic loci for STR profiles currently available in public databases.

## Results

### Incidence and extent of cell line cross-contaminants

**Fig 1:**
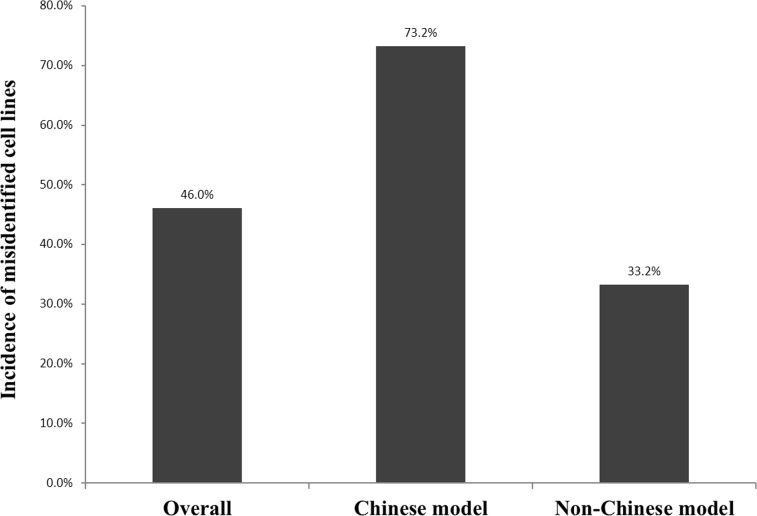
Incidence of misidentification among cell lines used in laboratories in China. 278 samples collected from 28 independent sources of China are divided into 2 groups according to their original source: Non-Chinese cell models (n = 193) and Chinese cell models (n = 71).

DNA from 278 cell lines was analyzed by STR profiling (S2 Table). By performing sensitivity evaluation assay for cross-contamination of intraspecies in the cell lines, we found that over 5% of the cell lines presented cross-contamination of intraspecies (Table 1). 20 samples (20/278 = 7.2%) that failed to be amplified by PCR were confirmed to be non-human origin. The success rate for complete analysis was 258/278 (92.8%). 150 cell lines were found to be unique (containing only one type of cells). The verified cell lines were derived from 20 different tissues (Table 2). Altogether, 128 instances of cross-contamination were confirmed, with an incidence of 46.0% (128/278). 84.4% (108/128) of cross-contamination was intra-species while 20 samples failed to be amplified by PCR and confirmed to be inter-species cross-contamination. We chose two of non-human cells to confirm the origin by species identification analysis [7], and confirmed that HIBEC cells were originally from rat and C201441 cells were from mouse (see S1 Fig). There were 14 cell lines for which we could not find any original documents about the cell line background, including C2013103, C201441, C201439, KI-H6, K324, A533, C201535, CB-06, D739, BC-034, CHMAS, PC-9, Anglne and CCLP-1. According to the original description, we divided the remaining cell lines (264 cell lines) into two categories: the Chinese model (cell lines established in China, n = 71) and the non-Chinese model (cell lines established outside China, n = 193) (Fig 1).

**Table 1:** Sensitivity evaluation of cell line intraspecies cross-contamination.

Ratio (ASPC-1: HeLa)	D19S433	D5S818	D21S11	D18S51	D6S1043	AMEL	D3S1358	D13S317	D7S820	D16S539	CSF1PO	Penta D	D2S441	vWA	D8S1179	TPOX	Penta E	TH01	D12S391	D2S1338	FGA
ASPC-1	14	12	28,30	18	11,20	X	16,	9,12	12,13	11	10,13	9,12	11,14	17	13,15	8,10	5,12	7,9.3	19	22,23	24
99:1	14	12	28,30	18	11,20	X	16,	9,12	12,13	11	10,13	9,12	11,14	17	13,15	8,10	5,12	7,9.3	19	22,23	24
95:5	13,14	11,12	27,28,30	16,18	11,18,20	X	15,16,18	9,12,13.3	8,12,13	9,10,11	9,10,13	8,9,12,15	10,11,14	16,17,18	12,13,15	8,10,12	5,7,12,17	7,9.3	19,20,25	17,22,23	18,21,24
90:10	13,14	11,12	27,28,30	16,18	11,18,20	X	15,16,18	9,12,13.3	8,12,13	9,10,11	9,10,13	8,9,12,15	10,11,14	16,17,18	12,13,15	8,10,12	5,7,12,17	7,9.3	19,20,25	17,22,23	18,21,24
80:20	13,14	11,12	27,28,30	16,18	11,18,20	X	15,16,18	9,12,13.3	8,12,13	9,10,11	9,10,13	8,9,12,15	10,11,14	16,17,18	12,13,15	8,10,12	5,7,12,17	7,9.3	19,20,25	17,22,23	18,21,24
HeLa	13,14	11,12	27,28	16	18	X	15,18	12,13.3	8,12	9,10	9,10	8,15	10,11	16,18	12,13	8,12	7,17	7	20,25	17	18,21

**Table 2:** The origin distribution of 150 unique cell lines.

Tissue	Liver	Colon	Bone	Lung	Peripheral Blood	Pancreas	Stomach	Cervix	Breast	Kidney
Number of instance	32	15	13	12	12	10	9	8	8	7
Tissue	Bladder	Ovarian	Skin	Prostate	Biliary tract	Bronchus	Umbilical vein	Ureter	Uterus	tongue
Number of instance	6	5	3	2	2	2	1	1	1	1

The authenticity test of non-Chinese cell model has been performed by cell repositories and their corresponding STR profiles that are available for comparison. 129 instances out of 193 characterized samples had distinct STR profiles and consisted of 78 individual cell lines. A total of 64 instances were unequivocally misidentified or cross-contaminated (64/193, 33.2%), including four examples that were notably false cell lines (U-251, Hep2, Ej and HBL-100) for which the actual identity had been previously reported [8–12], contributing 6.3% (4/64) of the contamination rate. The remaining 60 contaminated cell lines, contributing an incidence of 93.7% (60/64), had authenticated prototypes deposited in cell repositories or research institutions worldwide.

Compared to the non-Chinese group, extremely high levels of contamination were found among cell lines established in China. In the Chinese model, only 19 instances out of 71 had distinct STR profiles (26.8%) and consisted of 11 individual cell lines (Table 3). The remaining instances were unequivocally misidentified with an incidence of 73.2% (52 out of 71), affecting 24 cancer cell lines.

**Table 3:** STR profiles of misidentified cell line established by Chinese researcher.

Cell line	D19S433	D5S818	D21S11	D18S51	D6S1043	AMEl	D3S1358	D13S317	D7S820	D16S539	CSF1PO	Penta D	D2S441	vWA	D8S1179	TPOX	PentaE	TH01	D12S391	D2S1338	FGA
LO-2^a^^1^	13,14	11,12	27,28	16	18	X	15,18	12,13.3	8,12	9,10	9,10	8,15	10,11	16,18	13	8,12	7,17	7	20,25	17	18,21
SGC-7901^a^^1^	13,14	11,12	27,28	16	18,19	X	15,18	12,13.3	8,12	9,10	9,10	8,15	10,11	16,18	12,13	8,12	7,17	7	20,25	17	21
HL-7702^a^^2^	13	11,12	27,28	16	18	X	15,18	13.3	12	9,10	10	8,15	11	16,18	12	12	7,17	7	20,25	17	18,21
THC-8307^a^	13	11,12	27,28	16	18	X	15,18	13.3	12	9,10	10	8,15	11	16,18	12	12	7,17	7	20,25	17	18,21
BGC-823^b^^1^	13	10,11,12	27,28	13	18,19	X	15,18	7,13.3	11,12	9,11	9,10,12	8,15	10,11	16,17, 18	12,14	12	7,10,16,17	7,9	17,20,26	17,24	18,21
BEL-7402^a^	13	12	27,28	16	18,19	X	15,18	7,13.3	11,12	9,11	9,10,12	8,15	10,11	16,17, 18	12,14	12	7,17	7,	20,25	17	18,21
MGC-803^b^^1^	13	10,11,12	27,28	13	18,19	X	15,18	7,13.3	11,12	9,11	9,10,12	8,15	10,11	16,18	12,14	12	7,10,16,17	7,9	20,26	17,24	18,21
QBC-939^a^	13,14	11,12	27,28	16	18	X	15,18	12,13.3	8,12	9,10	9,10	8,15	10,11	16,18	13	8,12	7,17	7	20,25	17	18,21
GES-1^a^	13,14	11,12	30	14	12,18	X,Y	16	11	8,9	11,12	10	12	10,14	14,15	10,14	9,12	7,12	6	18,20	21,23	23,25
MGC-803^b^^2^	13	10,11,12	27,28	13	18,19	X	15,18	7,13.3	11,12	9,11	9,10,12	8,15	10,11	16,17,18	12,14	12	7,10,16,17	7,9	17,20,26	17,24	18,21
BGC-823^a^^2^	13.2,16	9,12	29	13	12	X	16	12	10,11	11,13	11,12	9,10	10,14	16,17	13	11,12	13,16	6,7	19,20	20,22	23,24
MGC-803^b^^3^	13	10,11,12	27,28	13	18,19	X	14,17	7,13.3	11,12	9,11	9,10,12	8,15	10,11	16,17,18	12,14	12	7,10,16,17	7,9	17,20,26	17,24	18,21
YTMLC-90^a^	13,14	11,12	27,28	16	18,19	X	15,18	12,13.3	8,12	9,10	9,10	8,15	10,11	16,18	12,13	8,12	7,17	7	20,25	17	21
SMMC-7721^a^	13	11,12	27,28	16	18	X	15,18,19	13.3	12	9,10	10	8,15	11	16,18	12	12	7,17	7	20,25	17	18,21
CCC-HIE2^a^	13	11,12	27,28	16	18	X	15,18,19	13.3	12	9,10	10,18	8,15	11	16,18	12	12	7,17	7	20,25	17	18,21
GIST-T1^a^	13	12	28,29,30	15	11,14	X	14,18	12,13	8,10	12,13	10	11,12	10,14	18	10,12	8	14,19	9	20,25	18	22,25
CNE2^b^^1^	13	11,12,13	30	13,16	11,14,18	X	15,18	10,12,13.3	10,12	9,10	10,11	9,12	11,14	14,16,17	12,13,17	8,9	17,20	6,7,9	20,21	17,23	18,21
BEL-7404^a^	13,14	11,12	27,28	16	18,19	X	18	12,13.3	8,12	9,10	9,10	8,15	10,11	16,18	12	8,12	16,17	7	20,25	17	21
BC-023^a^	15,18	8	28,30.2	17,18	11	X	15,17	12,14	11,12	9,13	12	9,10	11,15	16,19	12,14	11	7,15	7,9.3	19,21	19	23
BC-024^a^^1^	13	11	29	14,17	11,13	X,Y	16	11	8,11	11,12	10,12	9	10,13	14	13,14	8,11	7,11	8,9.3	18	24	23
BC-024^a^^2^	13	11	29	14,17	11,13	X,Y	16	11	8,11	11,12	10,12	9	10,13	14	13,14	8,11	7,11	8,9.3	18	24	23
HL-7702^a^^3^	13	11,12	27,28	16	18	X	15,18	13	12	9,10	10	8,15	11	16	12	12	7,17	7	20	17	18,21
ACC-2^b^	13	11,12	27,28	13,16	11,14,18	X	15,16,18	10,12,13.3	10,12	9,10	10,11	9,12	11	14,16	12,17	8,12	17,20	6,7,9	20,21	17,23	18,21
ACC-M^b^	13	11,12	27,30	13,16	11,14,18	X	15,16,18	10,12,13.3	10,12	9,10	10,11	9,12	11	14,16	12,16,17	8,12	16,17,20	6,7,9	20,21	17,13	18,21
CNE2^a^^2^	13,14	11,12	27,28	16,17	18	X	15,18	13.3	8,12	9,10	9,10	8,15	10,11	16,18	12,13	8,12	7,14	7	20,25	17	18,21
CEN2^a^^3^	13	11,12,13	30,32.2	13,19	11,14	X	15,16	8	9,11	11	11	11	11,13	18,19	16,17	8	12,19	7,9	18,20	21,24	18,21
CHMAS^a^^c^	14	12	29,30	14,15	11,12	X	15,16	8,11	11,12	11	13,14	10,12	10,14	16	12,13	8,11	13,14	7	18,20	17	22,24
TSCCA^a^	13,14	11,12	27,28	16	18,19	X	15,18	12,13.3	8,12	9,10	9,10	8,13	10,11	16,18	12,13	8,12	7,17	7	20,25	17	21
SGC-7901^a^^2^	N/D																				
MHCC-97H^a^	N/D																				
FRH0201^a^	N/D																				
BC-025^a^	N/D																				
BC-021^a^	N/D																				

^a^ The original cell line was replaced by another cell line; there was only one kind of cell in the cell line.

^b^ There were two kind of cell in the cell line.

^c^ original description could not been found.

^1,2,3^ for a single cell line from different sources. N/D: Non-detectable.

The amelogenin locus on the X and Y chromosomes were used for gender identification. Since Y chromosome was unstable, some cell lines and even normal cells from older male donors lost the Y chromosome [13], such as A549 and Jurkat cell line. Hence, an AMEL-X genotype did not confirm that the donor was female. However, the presence of a Y signal was only observed in cell lines derived from male donors. 44 out of 150 cell lines were males (29.3%). 3 cases of isogenic cell line pairs were actually derived from different tumor sites of the same patient. 95 C was obtained from the primary tumor site while 95 D originated from lymph nodes of the metastatic site. Similarly, Zos and Zos-M were established respectively from the primary tumor site and the skip metastasis of an osteosarcoma patient [14]. The pairwise comparison of STR profiles revealed three matched pairs with similarity scores >0.9. MHCC-97L, MHCC-97H and HCCLM-3 were in fact the same cell line. HCCLM-3 was a tumorigenic subclone of MHCC-97L and MHCC-97H, and was established from nude mice [15].

### Most common contaminants

In most instances (89/128), the cross-contaminating intruder could be identified via comparison in the databases provided by cell banks. The remaining 39 cell lines could not be identified by source. Cross-contamination of cell lines causes mischaracterization, which has been demonstrated in several studies. HeLa [16] and its derivatives HeLa S3, AV3 [17] and WISH contaminations are notorious problems and may be simply detected by either isoenzyme [18] or STR profiling [19]. Our findings showed evidence of 46.9% (60/128) cross-contamination was caused by HeLa, affecting 31 cell lines, which were purported to have been established from 10 types of tumor (colon, liver, breast, stomach, lung, nasopharynx, tougue, laryngeal, salivary gland and bronchia) and 3 types of normal tissue (lung, intestine and liver). The bladder cancer cell line T24 was established in 1972 and has contributed to several cell line cross-contamination [20, 21]. We identified three separate instances by T-24 in two laboratories, which might have used the established cell lines: LNCaP and EJ. Two multilocus profiles revealed a close relationship between LNCaP and EJ, which was a vector for T24 cell line. More details are shown in S3 Table.

**Fig 2:**
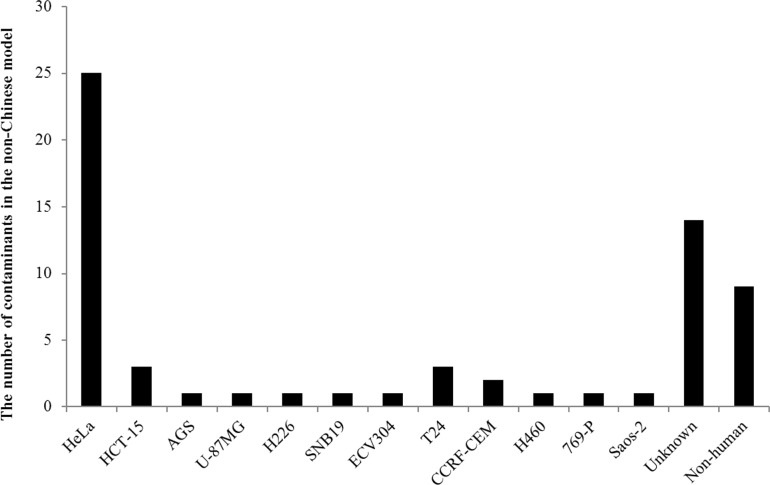
The numbers of cross-contamination in the non-Chinese model.

**Fig 3:**
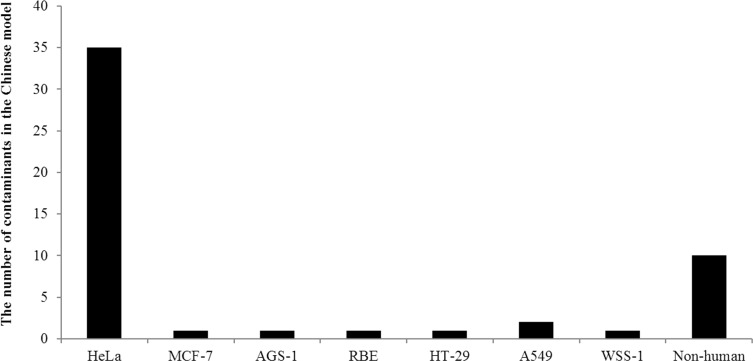
The numbers of cross-contamination in the Chinese model.

The prolific contaminants varied in each group. 25 instances of 64 contaminants in the non-Chinese model were HeLa, with an incidence rate of 39.1%. Contamination caused by non-human cell lines contributed a minor proportion (n = 9, 14.1%). The remaining contaminants identified in the non-Chinese model are described as follows: HCT-15 (n = 3), AGS (n = 1), U-87MG (n = 1), H226 (n = 1), SNB19 (n = 1), ECV304 (n = 1), T24 (n = 3), CCRF-CEM (n = 2), H460 (n = 1), 769-P (n = 1), Saos-2 (n = 1) and unknown (n = 14) (Fig 2). In contrast, contaminants found in the Chinese group were attributable to a few cell lines. Out of 52 identified contaminants, 35 were caused by HeLa with an incidence rate of 67.3%. Two adenoid cystic carcinoma, ACC-2 and ACC-M were reported as being contaminated by HeLa [22]. CNE-2, derived from the Chinese patients, was reported bearing HPV sequences and suspected to be contaminated by fusion of HeLa with another nasopharyngeal cell line [23]. Different samples of SGC-7901, SMMC-7721, BEL-7402, BEL-7404, BEL-7704, HL-7702, BGC-823 and TSCCA cell lines collected from independent laboratories were contaminated by either HeLa or another contaminant, possibly caused by a second intruder when they were cultured (Table 3). Contamination caused by non-human cell lines contributed 19.2%. The remaining contaminants found in the Chinese model are described as follows: MCF-7 (n = 1), AGS-1 (n = 1), RBE (n = 1), HT-29 (n = 1), A549 (n = 2), and WSS-1 (n = 1) (Fig 3).

### Genetic instability *in vitro*

In general, 69 HeLa cell lines showed one or more alterations in STR profile compared with the prototype cell. The differences among most of the samples from different laboratories were likely attributable to different culture conditions or passage number. This suggested HeLa cell line had remarkable degree of genetic instability as defined by STR profile. In sharp contrast, the subclones generated from K562 showed a high concordance of the clonotype homogeneity, i.e., one different clonotype was observed among the six K562 cell lines and the profile of the subclones differed from the parental culture by a single change of STR.

Comparison of original cell lines with the sublines derived from them revealed that nine other cell lines including 293, BxPC-3, HT-29, DLD-1, HCT-116, Jurkat, Raji, HeLa and 5637, exhibited variable STR profiles after multiple passages in culture. The subclones of these nine cell lines shared 80–94.12% identity to the original cell lines and appeared to bear null allele and mutations (Table 4). Several studies have demonstrated that Jurkat cell line was genetically unstable and the STR profile varied slightly among samples. One allele (Y) was missing in the locus amelogenin (X, Y and X were found in different subclone cell lines in all the cell banks) when compared to the published data. However, another locus D7S820 had a different allele when compared to our data. This discrepancy could be resulted from either a technical issue in PCR, in which one allele was preferentially amplified while another was below the detectable level, or a biological variation where the Jurkat cell line from one laboratory had lost one allele. In overall, all three cell lines should be considered as Jurkat.

**Table 4:** Representative data for comparison with ATCC.

Cell line	Resources	D5S818	D13S317	D7S820	AMEL	TPOX	D16S539	vWA	TH01	CSF1PO
293	ATCC	8,9	12,14	11	X	11	9,13	16,19	7,9.3	11,12
293	CCTCC	8,9	12,14	11,12	X	11	9	16,19	9.3	11,12
BxPC-3	ATCC	11	11	10,13	X	8	9,11	14,18	9	13
BxPC-3	CCTCC	11	11	10,13	X	8	11	14,18	9	13
Jurkat	ATCC	9	8,12	8,12	X	8,10	11	18	6,9.3	11,12
Jurkat	CCTCC	9	8,12	7.3, 12	X	8,10	11	18	6,9.3	11,12
HeLa	ATCC	11,12	12,13.3	8,12	X	8,12	9,10	16,18	7	9,10
HeLa	CCTCC	11,12	12,13.3	8,12	X	8,12	10	16,18	7	10
Raji	ATCC	10,13	13	10	X,Y	8,13	8,11	16,19	6,7	10,12
Raji	CCTCC	10,13	13	10	X,Y	8,13	8,11	17,19	6,7	10,12
HCT116	ATCC	11,10	12,10	11,12	X,Y	8,9	11,13	17,22	8,9	7,10
HCT116	CCTCC	11,10	12,10	10,11,12	X	8	11,13,14	17,23	8,9	7,10
HT-29	ATCC	12	11	10	X	8,9	12,13	17	6	11,12
HT-29	CCTCC	11,12	11,12	11	X	8,9	11,12	17,19	6	11,12
DLD-1	ATCC	13	8,11	10,12	X,Y	8,11	12,13	18,19	7,9.3	11,12
DLD-1	CCTCC	13	8,11	10,12	X,Y	8,11	12,13	18,19	7,9.3	12
5637	ATCC	11,12	11	10,11	X,Y	9	9	16,19	7,9	11
5637	CCTCC	11,12	11	10,11	X,Y	8	9	18	7,9	11

In contrast to the loss of heterozygosity, microsatellite instability [24], characterized by the occurrence of new alleles in tumors, is a major event in certain carcinomas brought about by mismatch repair deficiency. The extra band originated from one of two alleles with the characteristic stutter at one or more repeat unit below the main signal and the new allele differed from the assigned ancestral allele by one repeat. Microsatellite instability (MSI) was reported in leukemia [25], gastric [26], ovarian [27], breast [28], and colon cancers [29]. A total of 32 cell lines with MSI were identified with three banding patterns, including 7 liver cancer cell lines, 8 cervical cancer cell lines, 2 ovarian cancer cell lines, 1 leukemia cell line, 3 stomach cancer cell lines, 4 breast cancer cell lines, 1 colon cancer cell line, 2 osteosarcoma cancer cell lines, 1 pancreatic cancer cell line, 1 kidney cell line, 1 lung cancer cell line and 1 lymph cancer cell line. Our data suggested that allelic alterations might occur in higher frequency in tumors with microsatellite instability such as cervical carcinoma and hepatocellular carcinoma, with an estimated prevalence of 22.5–25%. The evaluation of our data revealed a tendency toward a correlation between microsatellite instability in tetra and penta markers. The most frequently implicated loci were observed in 32 tumors cell lines, included D3S1358, FGA, D12S391, D7S820, Penta D, Penta E, D18S51, D21S11 and D6S1043. On the other hand, vWA, D19S433, D16S539 and D13S317 loci were the least involved, showing three alleles at one locus. 32 cancer cell lines harbored novel alleles involving more than 50% of the 21 STR loci. We also observed that the mutation rate of markers fluctuated from 0.3% (D16S539) to 1.9% (D12S391) for HeLa and JVM-2 cell lines. Six cell lines exhibited hemizygosity at the loci on different chromosomes, affecting 3 alleles at 2 loci in Huh-7, SK-OV-3, HGC-27, Jurkat, Kato-III and HCT-116.

### Identity of HCCC-9810 and Calu-6

High degree of similarity (88.9%) was observed in one pair of cell lines, HCCC-9810 and Calu-6, by using ATCC STR database (9-loci STR database). This percent match indicated that these two cell lines were from the same origin. The HCCC-9810 cell line shared 4 same loci with the Calu-6 cell line at AMEL, D5S818, D13S317 and TH01, and differed from the Calu-6 cell line at other 5 loci by using the ATCC 9-loci STR database for comparison. However, when comparing these two cell lines by using 21-loci STR, we found that they still shared the same 4 loci as found by using 9-loci STR comparison, while the remaining 17 loci were different. The alleles of D21S11, D3S1358, D2S441, Penta E, D12S391 and FGA were totally different. When the same algorithm as used in 9-loci was adopted in 21-loci, the percent match of these two cell lines was 48.2%, indicating that they were from different origin (Table 5). To confirm the relationship between HCCC-9810 and Calu-6, direct sequencing of SNP was performed (Table 6). The SNP genotyping at 24-loci showed that there were 8 mismatches loci between HCCC-9810 and Calu-6 cell line, suggesting that they were from different origins [30].

**Table 5:** 21- STRloci profile for HCCC-9810 and Calu-6 cell lines.

Cell line	AMEL*	D5S818*	D13S317*	TH01*	D16S539*	CSF1PO*	vWA*	TPOX*	D7S820*	D19S433	D21S11	D18S51	D6S1043	D3S1358	Penta D	D2S441	D8S1179	Penta E	D12S391	D2S1338	FGA
HCCC-9810	X	11	11	9	**9**,12*	11*,12	16*,17	8,11*	10,12*	13,14*	**30**,32*	15*	19	17*	**9**,13	12*	10,13*	19*	19*	17,19*	**23**,24*
Calu-6	X	11	11	9	13*	12	17	8	10	13	31*	**12**,16*	13*,19	16*	13	**13**,14*	10,14*	**5**,14*	24*	17,26*	22*
Percent match	**88.9%**																				
	**48.2%**																				

*: Bolded loci were the 9-loci provided in ATCC and DSMZ databases. The difference of STR data between HCCC-9810 and Calu-6 were showed in bolded letters.

**Table 6:** 24 Loci SNP genotype profile for HCCC-9810 and Calu-6 cell line.

Designation	HCCC-9810	Calu-6
rs5742909	C	C
rs231775	**G**	**A**
rs1805010	**G**	**A**
rs1554606	G	G
rs2234767	G	G
rs1800682	T	T
rs1800869	G	G
rs1800872	**A**	**C**
rs1801275	A	A
rs1805015	T	T
rs5743836	T	T
rs3775291	G	G
rs3761623	A	A
rs3788935	G	G
rs352139	G	G
rs352140	**T**	**G**
rs8177374	C	C
rs9344	**G**	**A**
rs5743305	T	T
rs1800797	G	G
rs7208422	A	A
rs1042522	**C**	**G**
rs1041981	**C**	**A**
rs1801270	**C**	**A**

## Discussion

We presented a panoptic survey of cell line cross-contamination among human tumor cell lines using molecular genetic methods. The STR profiling technique uses fluorescence and multiplex PCR based technology to detect STR loci with overlapping size range. The specificity and polymorphism advantages make it method of choice for the authentication of human cell lines.

The misidentification and cross-contamination of cell lines are deemed to be a widespread problem in research, dating back as far as 45 years ago. Our lab started using STR for cell line authentication in 2009 with 16-STR loci. An alarming rate of cross-contamination among human cell lines used in China was reported by Fang Ye and colleagues [6] who investigated 380 human cell lines in which about 25% was cross-contaminated or misidentified. This study identified authentication problems with 46.4% of the cell lines that were analyzed and all the data were collected from 2014–2016, which were different from Fang Ye’s data. A total of 128 instances of cell line cross-contamination were detected among 278 original human cell lines. Our finding suggests that the relative contribution of cell line cross-contamination has been 1.3 times higher than that found in a previous study [31]. The simplest form of misidentification was mislabeling of cell line, which could be identified if cell line was profiled regularly. The detection sensitivity for cross-contamination by a small number of contaminating cell lines was 5%, which was in concordance with our report. Therefore, regular cell line authentication could be a straightforward way of catching cell line cross-contamination early.

Undue success in cell line cultures from primary material provides valid ground for suspicion of cell line cross-contamination. Normal diploid cell lines, namely CCC-HIE-2, FHC and FSH-74int, established by the Chinese Academy of Medical Sciences [32, 33], respectively, had been shown to be actually derived from HeLa cells. Instructively, spontaneous immortalization is rare in human cell lines. The HaCaT [34], HUVEC [35], SV-HUC-1[36], and hEM15A cell lines established from immortalized human skin cutis cells, umbilical cords, ureteral epithelium and endometrial stromal cells were totally free from cross-contamination.

Close to 67% of the misidentified cell lines established in China were contaminated by the HeLa cell line. In addition to their use as research tools, false or misidentified cell lines such as HL-7702, TSCCA and BEL-7402 have been used in anti-cancer drug screening programs in respected centers in China. WISH, which has been reported as a HeLa contaminant for decades [37], was also on the panel of cell models for differentiation [38] in these institutions under its false identity-amnion cell line of human origin. Despite sporadic reports about cell line cross-contamination provided by cell repositories in China through systematic quality control check of their own cell lines, the tainted cell lines are still on sale for use. It is hard to estimate how much research work is developed on false premise.

The grave problem of cell line misidentification in China has been neglected. Lack of information has prevented researchers from keeping up to date with literature reports of cell line misidentification. Although the international cell repositories have compiled a list of cross-contaminated cell lines to alert their users, the grave problem continues to fester due to the negligence of cell line users and originators as well as journal editors and funding agencies. Making the Chinese researchers aware of the situation is the most essential step. For those scientists working with cell lines, basic authentication test such as STR profiling, isoenzyme analysis, cytochrome C oxidase subunit I (COI) barcoding and mycoplasma contamination tests should be carried out and transferring cell lines to colleagues should be avoided.

STR-based profiling methods have been used for the authentication of cell lines for more than 24 years. However, there are variations in the numbers of loci and in the specific loci comprising the published profiling. We observed that HCCC-9810 and Calu-6 did not reliably discriminate between same or different origin within 9 markers. Obviously, authentication was greatly improved when we employed the 21-loci STR profiling and SNP genotyping method. Over the last years, several STR profiling assays have been published for developmental validation of new STR system. Compared to the current STR profiling, which included larger number of strategically selected markers for the human population, the discrimination power of 9 STR markers is satisfactory. Obviously, authentication was much more improved when we employed the 21-loci STR profiling and SNP genotyping method. Unfortunately, currant STR databases such as ATCC, DSMZ and JCRB only provide 9-loci STR profiles.

CCTCC (www.CCTCC.org) appealed to the scientific community to bring attention to the importance of cell line authentication. In 2016, a group of cell biologists led by CCTCC developed a program for cell line authentication. CCTCC repository generated a new 21-loci STR database (www.CCTCC.org/STR.html)). It was designed to represent a unique reference for validated molecular authentication of human cell lines, using the 21-marker commercial kit. End users could compare their STR profiles of cell lines from the website. The schema of the database included data tables, which consisted of a general table, where information on STR loci value could be entered, and match criteria have been set to 50–100% at 5% interval in accordance with ATCC, DSMZ and CCTCC standards. We hope that the verified cell lines available in CCTCC STR verification-database can strengthen the reproducibility and comparability of cell lines in different laboratories.

In conclusion, we authenticated a panel of 278 cancer cell lines. A high rate of cross-contamination (46.4%) emphasized the need for careful and frequent authentication of cell lines, possibly by more than one method. STR-based profiling method has been a great benefit to the scientific community, which has allowed cell line-based studies to be carried out without false premise resulted from misidentified or contaminated cell lines. To prevent exacerbation of the problem and reduce the frequency of cell-line misidentification and cross-contamination, the whole scientific community needs to get involved.

## Materials and Methods

### Cell lines

The authenticity of 278 tumor cell lines deposited in CCTCC by the 28 institutes was examined. The individual cell lines were supplied with duplicate, triplicate and quadruplicate collections from an independent source. The cell lines were grown in respective media at 37°C in a humidified incubator filled with 5% CO_2_.

### DNA extraction and STR profiling

DNA was isolated from cells using a genomic DNA purification kit (Tiangen Biotech, Beijing, China). Mutiplex PCR reactions were performed on a T-Gradient thermal cycler (Biometra, Gottingen, Germany). Briefly, DNA from each cell line was amplified using the Microreader TM21D System (Suzhou Microread Genetics, Beijing, China) according to the manufacturer’s instructions. The TM21D system uses primers to co-amplify 20 STR loci (including 13 combined core STR loci of DNA index system, namely CSF1PO, D3S1358, D5S818, D7S820, D8S1179, D13S317, D16S539, D18S51, D21S11, FGA, TH01, TPOX, vWA, and 7 other loci, namely Penta D, Penta E, D19S433, D16S1043, D2S441, D12S391, D2S1338) and amelogenin. The reaction mixture contained 2 ng genomic DNA, 10× PCR buffer, 2.5 mM dNTPs, 0.2 μM of each primer, and 2.5 U of Taq DNA polymerase. The PCR protocol was as follows: denaturing at 94°C for 5 min, denaturing at 94°C for 30 s, annealing at 60°C for 60 s, extension at 70°C for 60 s in 30 cycles and final extension at 60°C for 30 min. One μl aliquot of amplicon was mixed with 7.5 μl HiDiformamide, (Applied Biosystems, CA, USA) and 0.5 μl ROX-500 internal-lane size standards. The mixture was denatured at 95°C for 5 min and placed on ice immediately for 5 min, finally added 1 μl allelic ladder (Suzhou Microread Genetics, Beijing, China) and loaded on a POP-7 polymer gel (Applied Biosystems, CA, USA) for electrophoresis. The samples were electrophoresed with the GeneScan program on the ABI 3130 Genetic Analyzer (Applied Biosystem, CA, USA). The amplicon sizes were determined using GeneMapper 3.2 (Applied Biosystems, CA, USA). Alleles were designated by comparison to the allelic ladder. Each sample was repeated at least twice to confirm the results. If the sample could not be amplified in three time repeats, it will be considered as non-human origin. For data comparison, well-characterized and validated reference data obtained from ATCC and DSMZ were used. The algorithm of ATCC STR database was used for STR profile comparison. Percent match = Number of shared alleles between query sample and database profile/Total number of alleles in the database profile. Cell lines with ≥ 80% match are considered to be related, derived from a common ancestry. Cell lines with < 55% match are considered to be not related. Cell lines with 55% ~ 80% match require further authentication of relatedness.

### Sensitivity evaluation of intraspecies cross-contamination

To test the sensitivity of detecting cross-contamination, AsPC-1:HeLa cell mixtures at fixed ratios of 99:1, 95:5, 90:10 and 80:20 were generated. DNA was extracted from the cell mixtures using the genomic DNA purification kit. DNA concentrations were determined by a NanoDrop 8000 spectrophotometer. Contaminated samples were created by mixing each 50 ng/μl sample into the other to create 20%, 15%, 10%, 5% and 1% contamination by volume.

### Species identification analysis

DNA was isolated from cells using a genomic DNA purification kit (Tiangen Biotech, Beijing, China). DNA was amplified by PCR using 10 species-specific primer sets designed to amplify a specific product, respectively. The primers for 10 species were listed in the S4 Table. A549 (human), PK15 (pig), RK13 (rabbit), A9 (mouse), RIN-m5F (rat), Vero (African green monkey), MDCK (dog), MDBK (bovine), CHO-K1 (Chinese hamster) and BHK-21(Syrian hamster) cell lines were used as positive controls. Water was used as negative control for PCR. The PCR products were electrophoresized in a 2% agarose gel and visualized with ethidium bromide under UV light. The animals involved and expected PCR product sizes are as follows; human (1800bp), pig (517bp), rabbit (151bp), mouse (150 bp), rat (317 bp), African green monkey (222 bp), dog (153 bp), bovine (272 bp), Chinese hamster (293bp) and Syrian hamster (1500bp).

### SNP genotyping

HCCC-9810 and Calu-6 cell lines were obtained from the cell bank at the Chinese Academy of Science. The genetic identification of HCCC-9810 and Calu-6 cell lines was further characterized by SNP genotyping using direct SNP sequencing and standard PCR method. The 24 selected tagging SNPs were rs5742909, rs231775, rs1800682, rs2234767, rs1800896, rs1800872, rs1805010, rs1805015, rs1801275, rs1554606, rs1800797, rs1041981 and rs7208422 in immune response genes, rs9344, rs1801270, rs1042522 in cell cycle genes, and rs8177374, rs5743305, rs3775291, rs3788935, rs3761623, rs5743836, rs352139 and rs352140 in innate response genes. Genotyping was performed on ABI Prism 3100 Genetic Analyzer in conjunction with Clustal software. Details of PCR primer sequences for SNP genotyping were listed in S1 Table.

## Supporting Information

S1 Fig Species identification for HIBEC and C201441. (A) Species screen for HIBEC cell line. (B) Confirmation of rat origin for HIBEC cell line. (C) Species screen for C201441 cell line. (D) Confirmation of mouse origin for C201441 cell line.
(TIF)

S1 Table Primer sequences for SNP genotyping assay. (XLSX)

S2 Table 21-STR loci profiles for 278 cell lines. (XLSX)

S3 Table Summary findings for the provenance of cross-contamination cell lines. (XLSX)

S4 Table The gene-specific primer sets for 10 species. (XLSX)
