# Supplementary figures and images for: Investigation of Cross-Contamination and Misidentification of 278 Widely Used Tumor Cell Lines
Source: PLoS One. 2017 Jan 20;12(1):e0170384. doi: 10.1371/journal.pone.0170384 (PMC5249119; doi:10.1371/journal.pone.0170384)

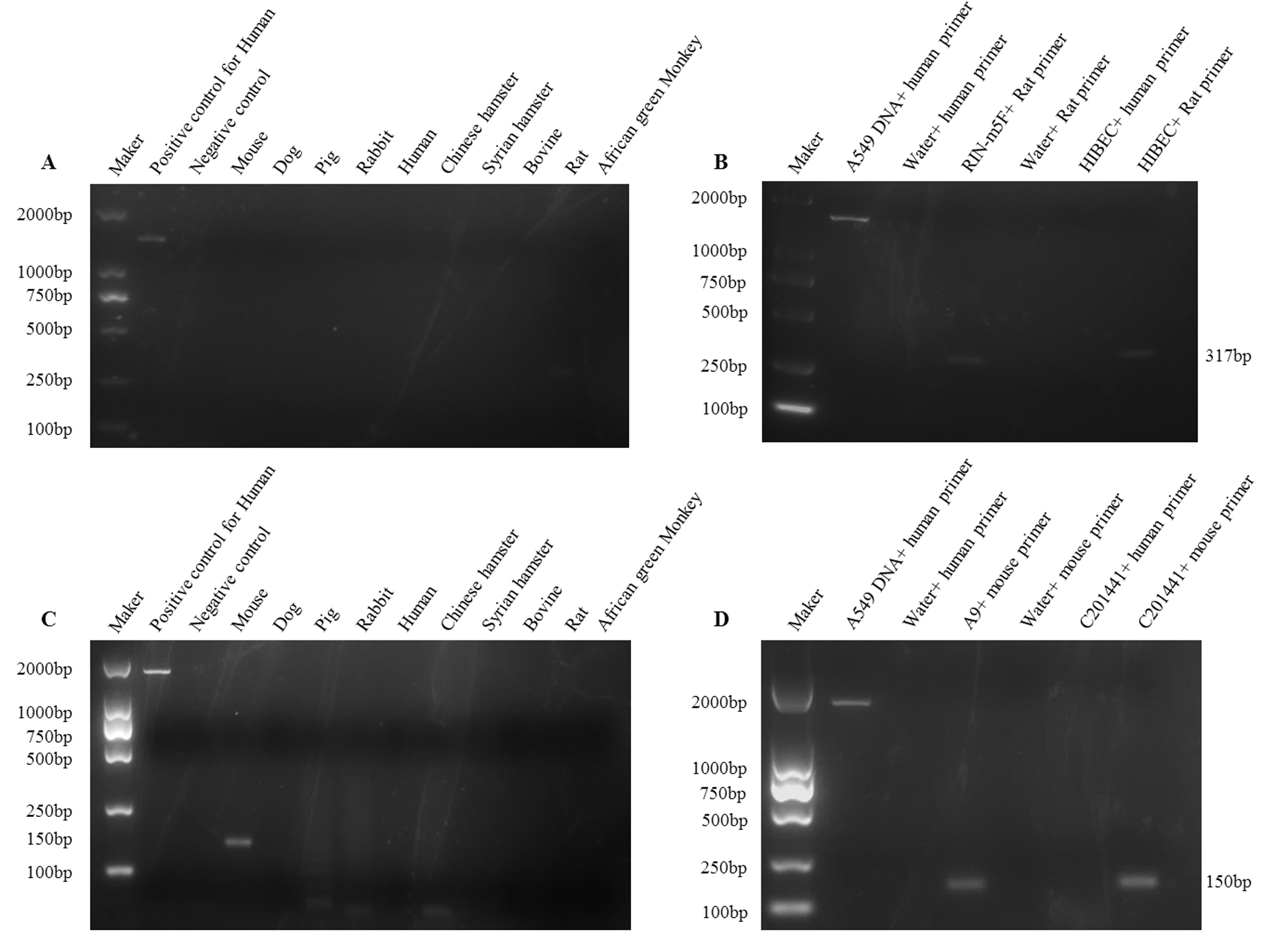

Supplement: S1 Fig — (A) Species screen for HIBEC cell line. (B) Confirmation of rat origin for HIBEC cell line. (C) Species screen for C201441 cell line. (D) Confirmation of mouse origin for C201441 cell line. (TIF) [file pone.0170384.s001.tif]
